# CT-defined muscle density as a prognostic factor in multiple myeloma undergoing autologous stem cell therapy: a retrospective single center study

**DOI:** 10.1007/s00432-024-06009-5

**Published:** 2024-11-15

**Authors:** Alexey Surov, Wolfram Pönisch, Jan Borggrefe, Hans-Jonas Meyer

**Affiliations:** 1 Department of Radiology and Nuclear Medicine, Ruhr-University of Bochum, Muehlenkreiskliniken Minden, Bochum, Germany; 2 Department of Hematology and Cell Therapy, University of Leipzig, Leipzig, Germany; 3 Department of Diagnostic and Interventional Radiology, University of Leipzig, Leipzig, Germany

**Keywords:** CT, Muscle density, Multiple myeloma

## Abstract

**Purpose:**

Skeletal muscle quality assessment can be performed by cross-sectional imaging. Skeletal muscle density (SMD) identified to be of prognostic relevance of several clinically outcomes in patients with hematological diseases. The purpose of the present study was to establish the effect of SMD on overall survival (OS) and progression-free survival (PFS) in patients with multiple myeloma (MM).

**Methods:**

All patients with MM were retrospectively analyzed between 2009 and 2019. 127 patients were included into the analysis. Whole-body computed tomography (CT) was used to calculate skeletal muscle index (SMI), SMD, albumin-gauge score and intramuscular adipose tissue content (IMAC).

**Results:**

Overall, 28 patients (22.0%) of the patient sample died. In the discrimination analysis muscle density was higher in non-survivors compared to survivors (mean 30.8 ± 12.5 versus 24.1 ± 15.8, p = 0.03) and IMAC was lower in non-survivors (− 0.66 ± 1.8 versus − 0.25 ± 0.21, p = 0.01). These differences, however, were not demonstrated in the logistic regression analysis, which could not show prognostic relevance for the investigated muscle density parameters on PFS or OS.

**Conclusion:**

CT-defined muscle density parameters have no prognostic relevance on survival in patients with MM undergoing autologous stem cell therapy, which was demonstrated in a comprehensive analysis. These results corroborate previous smaller studies that body composition might have a limited role in this tumor entity.

## Introduction

Computed tomography (CT) defined body composition can provide several image-defined prognostic factors (Wang and Bai 2012; Janssen 2011; Prado et al. 2008; Fielding et al. 2011; Jang et al. 2020; Chianca et al. 2021). Among these, skeletal muscle area (SMA) is a surrogate parameter for muscle quantity, whereas skeletal muscle density (SMD) is a surrogate parameter for muscle quality. It has been previously demonstrated that skeletal muscle characteristics defined by CT images are directly associated with several clinically relevant outcomes in oncology patients, which has been discussed in relation to frailty and muscle wasting (Wang and Bai 2012; Janssen 2011; Prado et al. 2008; Fielding et al. 2011; Jang et al. 2020; Chianca et al. 2021; Pinto et al. 2022; Aleixo et al. 2020 Jan; Jogiat et al. 2022 Dec; Takeoka et al. 2016).

In the fields of oncology and hematology, the prognostic importance of imaging defined body composition of low skeletal muscle mass and low SMD has been demonstrated with high evidence for several different tumors (Wang and Bai 2012; Janssen 2011; Prado et al. 2008; Fielding et al. 2011; Jang et al. 2020; Chianca et al. 2021; Pinto et al. 2022; Aleixo et al. 2020 Jan; Jogiat et al. 2022 Dec; Takeoka et al. 2016).

In multiple myeloma (MM), the association between low-skeletal muscle mass as the surrogate parameter for sarcopenia has been investigated previously in several studies with heterogeneous, inconclusive results (Takeoka et al. 2016; Cunha Júnior et al. 2021; Umit et al. 2020 Nov; Tagliafico et al. 2022 Apr 1; Nandakumar et al. 2023 Feb 1; Surov et al. 2023 Dec). Some authors demonstrated a prognostic relevance (Cunha Júnior et al. 2021; Nandakumar et al. 2023 Feb 1), whereas the majority of studies failed to show a statistically significant association of low skeletal muscle mass with the overall survival (Takeoka et al. 2016; Surov et al. 2023 Dec). These results may be explained by the different imaging methods used, such as PET-CT, whole-body CT or contrast enhanced staging CT, which may also lead to different results for body composition parameters (Takeoka et al. 2016; Cunha Júnior et al. 2021; Umit et al. 2020 Nov; Tagliafico et al. 2022 Apr 1; Nandakumar et al. 2023 Feb 1; Surov et al. 2023 Dec).

However, the assessment of body composition in patients with MM may be critical as these patients are at high risk for anorexia, malnutrition, and muscle wasting (Zweegman et al. 2017; Fraz et al. 2019 May; Patel et al. 2020 Feb). In addition, frailty and potential fracture risk are of paramount importance, as these patients may frequently develop pathologic osteolysis (Wang et al. 2019; Nielsen et al. 2018). Notably, the association between low skeletal muscle mass and fracture risk has been demonstrated in large analyses of patients with osteoporosis (Wang et al. 2019; Nielsen et al. 2018).

Another important aspect is that MM patients undergo regular staging CT scans to exclude or diagnose new osteolysis, which could also be used as a longitudinal parameter.

Notably, previous studies regarding MM only evaluated the muscle quantity defined by skeletal muscle area but not the muscle quality defined by SMD (Takeoka et al. 2016; Cunha Júnior et al. 2021; Umit et al. 2020 Nov; Tagliafico et al. 2022 Apr 1; Nandakumar et al. 2023 Feb 1; Surov et al. 2023 Dec). This imaging parameter may provide more prognostic information regarding the general constitution of the skeletal muscle status of the patients, which needs to be elucidated in a systematic analysis.

Therefore, the aim of the present study was to evaluate the associations between muscle quality parameters derived from CT images with overall and progression-free survival in MM patients undergoing autologous stem-cell therapy (aPBSCT).

## Methods

### Patient acquisition

This single-center, retrospective observational study was approved by the institutional review board (IRB 00001750; registration number 118/18-ek).

All patients with MM, who underwent aPBSCT between 2009 and 2019 were retrospectively included. In every case, an aPBSCT was performed in a curative intent. Throughout this time frame, bortezomib was used as an induction therapy.

A total of 127 patients (50 female patients, 39.4%) with a mean age of 57.8 ± 7.6 years with sufficient clinical and imaging data were identified in the data base.

### Clinical parameters

The following clinical parameters were obtained from the patients' medical records at the time point of the diagnosis: blood cell count; the serum levels of C-reactive protein, lactate dehydrogenase (LDH), b2-microglobulin, creatinine, albumin, and calcium; creatinine clearance as a factor of renal insufficiency; quality and quantity of M protein in blood and urine samples; clinical stage according to the Salmon and Durie classification and International Staging system (ISS); cytogenetics: autologous peripheral blood stem cell transplantation (aPBSCT); the disease progression was evaluated according to the guidelines of the International Myeloma Working Group (Rajkumar et al. 2014); death and overall survival.

### Imaging technique

CT imaging was performed on a 128-slice or 256-slice clinically used CT scanner (Ingenuity or iCT256, Philips, Hamburg, Germany). The CT investigation at diagnosis was used for the body composition calculation. The used imaging parameters were 120 kVp, 36 mAs, collimation of 64 × 0.6 mm and pitch of 0.8. The scan length included the following body regions: head, neck, chest, abdomen/pelvis, upper limb and the proximal half of the lower limb. The minimal slice thickness was 1 mm.

### Skeletal muscle measurements

Body composition parameters were quantified semi-automatically using ImageJ software 1.48v (National Institutes of Health Image program). Skeletal muscle areas were calculated at the L3 level including the psoas and paraspinal muscles. The muscle area was identified using the HU thresholds of -29 and 150 HU, as proposed in similar studies (Chianca et al. 2021). Skeletal muscle area was divided by height to calculate the skeletal muscle index (SMI). Skeletal muscle density (SMD) was defined in HU. Intramuscular adipose tissue content (IMAC) was measured by dividing the SMD by the HU values of the subcutaneous tissue as reported in the study by Hamaguchi et al. (Hamaguchi et al. 2014 Nov). A lower IMAC indicates skeletal muscle with little adipose tissue, a higher IMAC indicates more adipose tissue within the muscle and therefore poorer muscle quality, while a lower IMAC indicates skeletal muscle with less adipose tissue and better muscle quality. The cut-off values proposed by Hamaguchi et al. of -0.32 for men and -0.13 for women were used to categorize the patient sample (Hamaguchi et al. 2014 Nov).

To define low skeletal muscle density, the median value of the current patient sample was used, in addition to the cut-off values proposed by Nachit et al. of 28.1 HU for males and 18.9 HU for females and Sjoblom of 28.0 HU for males and 23.8 HU for females. As proposed in recent studies as a promising combined prognostic marker, the albumin gauge score was calculated as the multiplication of serum albumin by SMD.

Finally, muscle-gauge was calculated as SMI multiplied by SMD (Tonnesen et al. 2023). Measurements were performed by a board-certified radiologist with 8 years of general experience who was blinded to the clinical outcome of the patients.

**Fig. 1:**
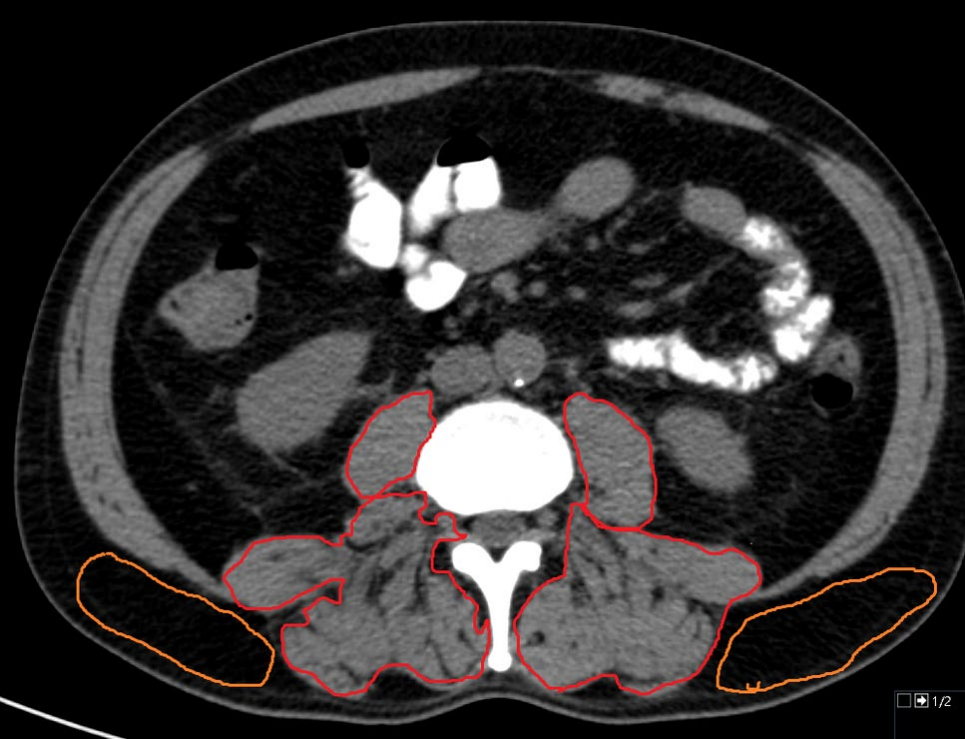
Representative patient of the patient sample. The region of interest is drawn to measure the skeletal muscle density of the paraspinal muscles (red). The subcutaneous fat area density was measured to calculate the intramuscular adipose tissue content (orange). The patient had low skeletal muscle density and high intramuscular adipose tissue content

Figure 1 shows a representative patient from the study to illustrate the CT measurements.

### Statistical analysis

The statistical analysis and graphics creation were performed with GraphPad Prism 10 (GraphPad Software, La Jolla, CA, USA) and SPSS package (IBM SPSS Statistics for Windows, version 225.0: IBM corporation). Data were expressed by means of descriptive statistics (absolute and relative frequencies). To test for group differences Mann–Whitney-U test and Fisher exact test were used when suitable. Spearman’s correlation analysis was used to elucidate possible associations between clinical parameters and muscle density parameters. Binary logistic regression analysis was used for associations between the muscle density parameters with overall and progression-free survival. For all tests, *p-v*alues < 0.05 were used to indicate statistical significance.

## Results

Overall n = 28 patients (22.0%) of the patient sample died; median 33 months range 4–396 months. All cases died from MM-related causes. Amyloidosis was the cause in 3 cases (10.7%), sepsis in 2 cases (7.1%), graft versus host disease in 4 cases (14%), and tumor burden in the remaining 19 cases (67.8%). The median PFS was 24 months (95% CI 20;30) and the median OS was 33 months (95% CI 28;40).

Table 1 provides an overview of the demographics of the investigated patient sample.

**Table 1:** Baseline characteristics, treatments, and causes of death

Parameter	N = 128, mean ± SD
Age (y)	57.8 ± 7.6
Gender (female, n, %)	50 (39.4)
Albumin (g/dL)	42.1 ± 32.3
Calcium (mmol/L)	2.4 ± 0.3
Beta-microglobulin (mg/L)	Median 3.5
Hemoglobin (g/dL)	10.5 ± 2.2
Creatinine clearance (mL/min)	68.9 ± 33.3
M-protein in urine (g/dL)	27.2 ± 23.8
Lactate dehydrogenase (U/L)	8.8 ± 37.8
ISS stage	I: n = 54, II: n = 35, III: n = 39
Salmon and Durie stage	Ia: n = 2, IIa: n = 6, IIIa: n = 89, IIIb: n = 31
Cytogenetic risk profile	High (n = 34, 26.6%), low (n = 80, 62.5%), not performed (n = 14, 10.9%)
Cytogenetic alterations	P53 deletion (n = 18), t4/14 (n = 16), t4/16 (n = 5), del 13 (n = 49), hyper (n = 10), del 1 (n = 25), t11/14 (n = 12)
Skeletal muscle density (HU)	25.6 ± 15.4
Skeletal muscle index (cm^2^/m^2^)	51.4 ± 9.2
Muscle gauge	1346 ± 822
Subcutaneous adipose tissue (HU)	− 100.1 ± 15.9
Intramuscular adipose tissue content	− 0.34 ± 0.9
Album-Gauge score	1021 ± 722.9

*SD* standard deviation, *ISS* international staging system, *HU* hounsfield unit

**Fig. 2:**
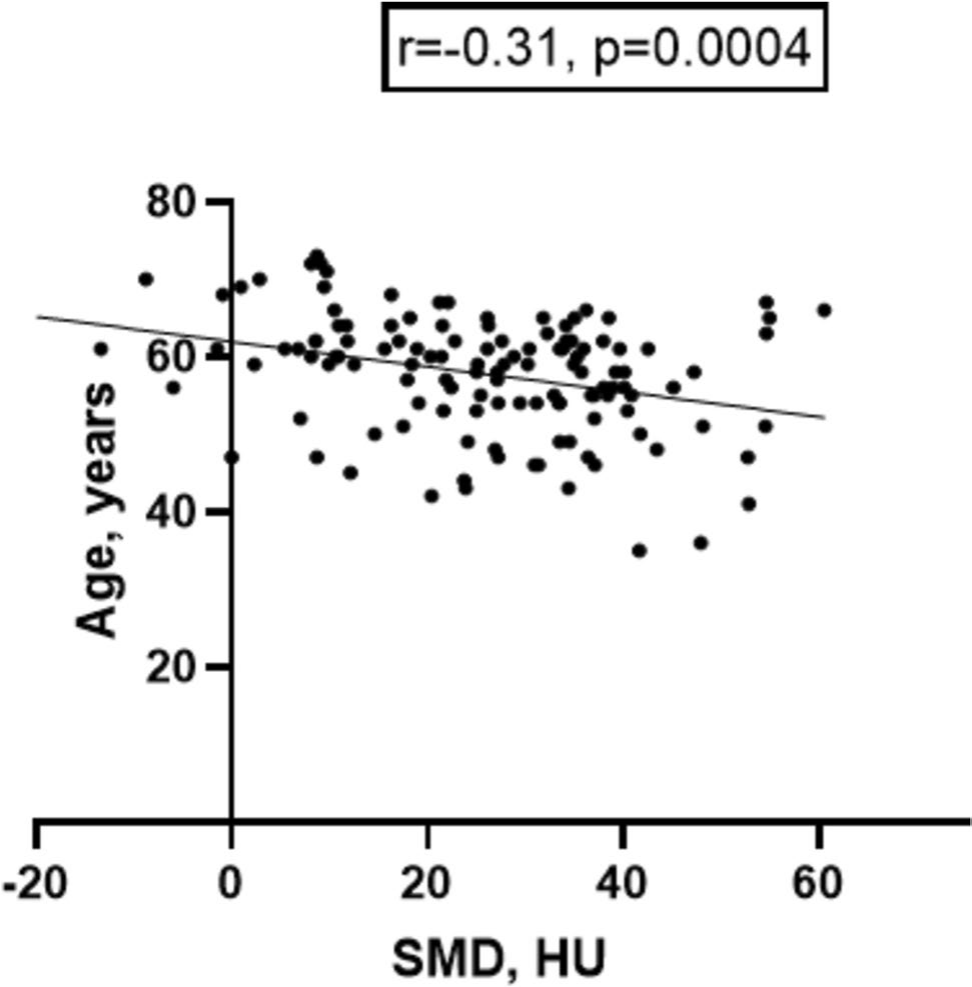
Spearman’s correlation analysis between age and skeletal muscle density. A weak inverse correlation is demonstrated (r = − 0.31, p = 0.0004)

The investigated skeletal muscle parameters differed between male and female patients. The SMD was slightly higher in male compared to female patients (27.6 ± 15.9 HU versus 22.3 ± 14.1 HU, p = 0.01). Similarly, SMI was higher in male patients (54.5 ± 8.2 cm^2^/m^2^ versus 44.8 ± 7.5 cm^2^/m^2^, p < 0.0001), whereas IMAC was lower in male patients (-0.40 ± 1.1 versus -0.24 ± 0.21, p = 0.04). Contrary, the density of the subcutaneous fat was not different (-100.6 ± 15.3 HU versus -99.1 ± 17.1, p = 0.50). SMD was inversely correlated with age of the diagnosis (r = -0.31, p = 0.0004, Fig. 2), whereas IMAC is positively correlated with (r = 0.29, p = 0.0006).

There were no correlations between SMD or IMAC with initial ISS stage at diagnosis (r = -0.05, p = 0.90 and r = 0.01, p = 0.86, respectively).

### Survival analysis

**Fig. 3:**
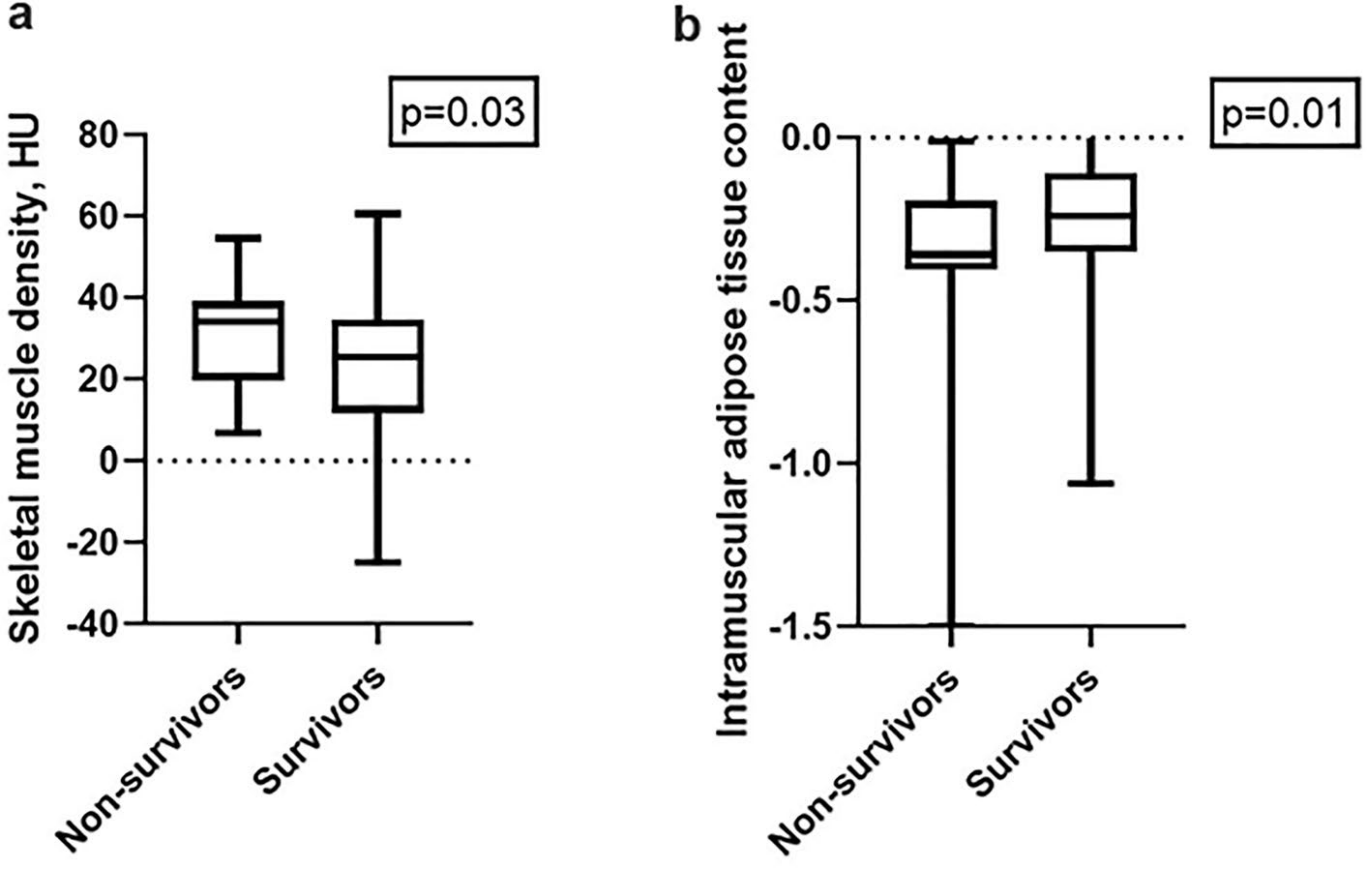
**A** Box plot graph of the skeletal muscle density between survivors and non-survivors. The non-survivor group had higher skeletal muscle density values with a p-value of 0.03. **B** Similar results were shown for the parameter intramuscular adipose tissue content with a p-value of 0.01

In the discrimination analysis muscle density was higher in non-survivors compared to survivors (mean 30.8 ± 12.5 versus 24.1 ± 15.8, p = 0.03) and IMAC was lower in non-survivors (-0.66 ± 1.8 versus -0.25 ± 0.21, p = 0.01) (Table 2). The other investigated parameters were not different between the groups. Figure 3 shows the corresponding box plot graphs.

**Table 2:** Comparison of skeletal muscle parameters according to survival

Parameter	Survivors (n = 99, M ± SD)	Non-Survivors (n = 28, M ± SD)	p-values
Skeletal muscle density (HU)	24.1 ± 15.8	30.8 ± 12.5	**0.03**
Skeletal muscle index (cm^2^/m^2^)	50.9 ± 9.6	51.9 ± 7.3	0.67
Muscle gauge	1320 ± 822.5	1578 ± 737	0.12
Subcutaneous adipose tissue (HU)	− 100.2 ± 16.4	− 99.5 ± 14.7	0.51
Intramuscular adipose tissue content	− 0.25 ± 0.21	− 0.66 ± 1.8	**0.01**
Low albumin (cut-off 48 g/L)	50 (50.5)	13 (46.4)	0.91
Low muscle density (cut-off median)	53 (53.5)	10 (35.7)	0.34
High intramuscular adipose tissue content	49 (49.5)	18 (64.3)	0.48
Albumin gauge score	984.4 ± 777.5	1151 ± 470.8	**0.04**

After division of the patient sample accordingly to gender, the statistical significance was not reached in the subgroups for male or female patients, respectively (Tables 3 and 4).

**Table 3:** Comparison of skeletal muscle parameters according to survival in female patients (n = 50)

Parameter	Survivors (n = 40, M ± SD)	Non-Survivors (n = 10, M ± SD)	p-values
Skeletal muscle density (HU)	29.0 ± 13.6	20.8 ± 13.9	0.13
Skeletal muscle index (cm^2^/m^2^)	44.6 ± 7.4	45.8 ± 8.0	0.74
Subcutaneous adipose tissue (HU)	− 99.2 ± 16.8	− 98.6 ± 19.3	0.88
Intramuscular adipose tissue content	− 0.23 ± 0.21	− 0.32 ± 0.22	0.15

**Table 4:** Comparison of skeletal muscle parameters according to survival in male patients (n = 77)

Parameter	Survivors (n = 57, M ± SD)	Non-Survivors (n = 18, M ± SD)	p-values
Skeletal muscle density (HU)	26.3 ± 16.8	31.6 ± 12.3	0.15
Skeletal muscle index (cm^2^/m^2^)	54.8 ± 8.7	53.3 ± 6.7	0.39
Subcutaneous adipose tissue (HU)	− 100.9 ± 16.2	− 99.8 ± 12.6	0.31
Intramuscular adipose tissue content	− 0.26 ± 0.20	− 0.82 ± 2.2	0.09

In the logistic regression analysis SMD as metric variable showed an association with HR of 1.03 (95%CI 1.001, 1.06, p = 0.044). The other parameters did not show prognostic relevance on progression-free or overall-survival (Tables 5 and 6). The collinearity test demonstrated low VIF values up to 1.03 demonstrating low correlation between the variables and therefore independence of each other. Exemplarily, SMD had a HR of 0.48 (95% CI 0.20; 1.11, p = 0.10) for overall survival and 0.50 (95%CI 0.17;1.46, p = 0.21) for progression-free survival.

**Table 5:** Logistic regression analysis for the effect of the investigated muscle density parameters on OS (Univariable Analysis)

Parameters	HR	CI 95%	p-values
Low muscle density (cut-off median)	0.48	0.20, 1.11	0.10
Skeletal muscle density (metric)	1.03	1.001, 1.06	0.044
Low albumin (cut-off 48 g/L)	0.73	0.31, 1.71	0.47
High intramuscular adipose tissue content (cut-off-0.32 for men and − 0.13 for women)	1.91	0.80, 4.55	0.14
High intramuscular adipose tissue content (metric)	0.24	0.031, 1.88	0.17
Low albumin/low muscle density	0.37	0.10, 1.35	0.13
High intramuscular adipose tissue content/low albumin	1.23	0.46, 3.30	0.67
Low skeletal muscle density defined by median	0.48	0.20, 1.1	0.10
Low skeletal muscle density defined by Nachit	0.74	0.31, 1.75	0.50
Low skeletal muscle density defined by Sjoblom	0.66	0.28, 1.54	0.34
Albumin-gauge score as metric variable	1.01	1.0, 1.02	0.30
Low albumin-gauge score defined by median 999.7	0.42	0.13, 1.32	0.14
Low albumin-gauge score defined by first quartile 535	0.67	0.29, 1.58	0.36

*HR* hazard ratio, *CI* confidence interval

**Table 6:** Logistic regression analysis for the effect of the investigated body composition parameters on PFS (Univariable Analysis)

Parameters	HR	CI 95%	p-values
Low muscle density (cut-off median)	0.50	0.17, 1.46	0.21
Skeletal muscle density (metric)	1.003	0.98, 1.02	0.81
Low albumin (cut-off 48 g/L)	0.70	0.25, 1.97	0.50
High intramuscular adipose tissue content (cut-off-0.32 for men and − 0.13 for women)	2.48	0.82, 7.53	0.10
High intramuscular adipose tissue content (metric)	1.39	0.60, 3.17	0.43
Low albumin/low muscle density	0.20	0.02, 1.59	0.12
High intramuscular adipose tissue content/low albumin	1.57	0.50, 4.92	0.43

*HR* hazard ratio, *CI* confidence interval

## Discussion

The present study investigated the prognostic relevance of CT-defined muscle quality parameters on OS and PFS in patients with MM undergoing autologous stem cell therapy. In short, the study could show some differences between survivors and non-survivors regarding skeletal muscle density, but this was not related to the OS and PFS analyses. Presumably, the patient sample of this single center study was too small to show a statistically significant effect of this body composition parameter on survival outcomes.

Body composition is an emerging area of research with extensive studies in several disease entities with overall promising results for additional risk stratification purposes provided by cross-sectional imaging (Wang and Bai 2012; Janssen 2011; Prado et al. 2008; Fielding et al. xxxx; Jang et al. 2020; Chianca et al. 2021; Pinto et al. 2022). Notably, sarcopenia and fat assessment have been shown to be prognostically relevant in several aspects of oncologic disease (Wang and Bai 2012; Janssen 2011; Prado et al. 2008; Fielding et al. xxxx; Jang et al. 2020; Chianca et al. 2021; Pinto et al. 2022). It should be emphasized that body composition assessment is a by-product of cross-sectional imaging that can be easily calculated and implemented into clinical routine without additional radiation exposure (Chianca et al. 2021; Pinto et al. 2022).

In particular, oncologic and hematologic patients are at risk for skeletal muscle wasting due to several factors, including prolonged bed rest and systemic inflammation (Zweegman et al. 2017; Fraz et al. 2019 May; Patel et al. 2020 Feb). In addition, MM patients often undergo whole-body CT or MRI (Rajkumar et al. 2014).

The importance of skeletal muscle area assessment in hematologic diseases was demonstrated in a recent meta-analysis (Surov and Wienke 2021 Mar). In the overall analysis, which pooled different hematologic diseases, sarcopenia was independently associated with lower OS with a reported HR = 1.94, CI 95% 1.30–2.90, p < 0.001 (Surov and Wienke 2021 Mar). However, most of the included studies included patients with lymphoma and leukemia, and only one study included patients with MM (Surov and Wienke 2021 Mar). Since then, more studies on MM have been published, but they have not yet been systematically pooled in another meta-analysis (Takeoka et al. 2016; Cunha Júnior et al. 2021; Umit et al. 2020 Nov; Tagliafico et al. 2022 Apr 1; Nandakumar et al. 2023 Feb 1; Surov et al. 2023 Dec). Takeoka reported negative results for the association between sarcopenia and overall survival in 56 patients (Takeoka et al. 2016). Similar results were published by Williams et al. in 142 MM patients (Williams et al. 2021 Jan).

In another recent study, da Cunha Júnior et al. evaluated 76 patients with MM using FDG-PET (Cunha Júnior et al. 2021). A key finding of this study was that visceral fat metabolic activity was strongly associated with overall survival with an HR of 13.36 (95% CI 3.12, 57.15, p < 0.001) in multivariate analysis (Cunha Júnior et al. 2021). However, skeletal muscle area and visceral fat area had no prognostic relevance for overall survival (Cunha Júnior et al. 2021).

However, in contrast to these studies, the prognostic relevance of muscle quality in MM patients is still unclear. This is important because in other disease entities, the superiority of muscle quality assessment over muscle surface quantity has been highlighted (Pinto et al. 2022). However, there are only few data available for hematologic diseases.

This is particularly controversial because in our patient cohort, surviving cases were found to have lower muscle density and consequently lower muscle quality than those who died. This is in contrast to other diseases where myosteatosis has been associated with a 75% higher mortality (Aleixo et al. 2020 Jan). The present contradictory results need to be clarified in other patient cohorts with MM.

It can be assumed that the change in muscle density throughout the patient's history may be more relevant than the status at the beginning of treatment.

Furthermore, it should be discussed that the identified HR of 2.48 for IMAC on PFS can be considered as an intermediate effect size, which probably did not reach statistical significance due to the small patient sample size (Flório et al. 2023 Feb).

The merit of the current study is the comprehensive investigation of muscle density cut-offs, which clearly demonstrated differences for prognostication. This study is the first to test the effect of different muscle quality cut-offs and to incorporate the effect of albumin as an important blood parameter with muscle quality defined by CT.

There are some statistical signals that the albumin gauge score might be relevant, which was not yet shown in the logistic regression analysis. Clearly, larger multicenter analyses are needed to harmonize the predominantly negative results regarding CT body composition in patients with MM.

Limitations of the present study must be acknowledged. First, the retrospective observational study design with possible inherent bias. Second, the patient sample is relatively small due to the single-center design. This also resulted in possible non-significance of our hazard ratios. In addition, the presented standard deviations are rather large, which is also an effect of the small sample size. Third, the measurements were performed by a single reader, which could lead to some reader bias. However, the region of interest was drawn in a standardized manner. Fourth, the time period of the study is rather long, which could lead to differences in survival due to potential differences in treatment. However, we could not further adjust for this fact.

## Conclusion

CT-defined muscle density parameters have no prognostic relevance on survival in patients with MM undergoing autologous stem cell therapy, which was demonstrated in a comprehensive analysis. These results corroborate previous smaller studies that body composition might have a limited role in this tumor entity.

## Data Availability

No datasets were generated or analysed during the current study.

## Abbreviations

CT: Computed tomography
SMD: Skeletal muscle density
MM: Multiple myeloma
aPBSCT: Autologous stem-cell therapy
SMI: Skeletal muscle index
IMAC: Intramuscular adipose tissue content
HR: Hazard ratio
ISS: International staging system
